# Sleeping with Others, Etc.

**Published:** 1862-06

**Authors:** 


### SLEEPING WITH OTHERS, ETC.

THE following case came under the Editor's attention:

"My little son, eight years old, is lively and well in all respects, as far as I can judge, in the day, but at night he wakes up and screams in the most distressing manner. He awakes frightened almost every night for a year. He has been subject to this for several years, but it is getting to be a distressing case. I almost dread to go to sleep. He sleeps with me, and once every night, sometimes twice, he springs as quick as lightning, screams the most soul-piercing screams, and I take him in my arms, and he looks back, with horror on his face. I could not, unless you saw it, give you an idea of how distressing it is. He is strong and lively, playful, and full of fun and life, until he gets to sleep. This happens every night, and often it so shocks me I can not go to sleep for a long time. He takes dinner at one, supper at sundown, of cambric tea with a piece of bread. All my other children are entirely different; they are lively, hearty, and cheerful, and so is he, otherwise."

The directions given were on the presumption that these effects in the child were caused by sleeping in the same bed with the father, habitually. In two months, the report was made that "he had not waked up frightened for a long time." The national troubles at this point put an end to the correspondence. It may be instructive to know under what bodily symptoms the father had been laboring for some years, but whom the author succeeded in restoring to "better health than had been enjoyed for seven years." Age, thirty-seven; hight, five feet seven; broad-shouldered, dark skin, black eyes, and had remarkable health, strength, and activity, never having had any severe sickness; began to chew tobacco ravenously, and became gradually very nervous, from one meal to another; especially in summer, would become so exhausted as to scarcely be able to walk. The least excitement or agitation would keep him from eating enough to sustain his strength until the next meal-time. Then came on chronic diarrhea, aggravated by any unusual intelligence, or shock of any description. "The least feeling like it, or appearance of loose bowels, completely unmans me; I can't help it; and if I was very hungry or exhausted, the occurrence of any looseness kills my appetite. I can not help being alarmed at any symptom of diarrhea, for it has used me up long ago. I am greatly depressed in spirits from the sympathy incident to planning for and nursing my children, and solicitude for them in sickness, for these ten years. My wife is an angel of goodness to me, and I have every reason to be a happy man. Please don't say any thing that might have a tendency to depress me. As soon as I get the least hungry, I become weak and nervous, and the same if there is any cause for anxiety or excitement. Coffee is poison to me; two swallows would distress my head and make my ears feel as if water were in them; at the same time there is a feeling of wanting to draw a long breath. If I talk much, or read aloud at times, I feel as if I had been violently blowing up a fire. Hot weather is ruinous to me." It was in August, when the thermometer had been ranging at one hundred degrees Fahrenheit, that he wrote of the great improvement in his health. This man's nervous system had been so impaired, and his general health so poor, that it is not to be wondered at if a young child sleeping with him constantly should have its own nervous organization impaired. On being required to sleep in another bed; to go to bed late, but regularly; to sleep on a pillow a little high, so as to antagonize the blood accumulating in the brain; to be waked up about fifteen minutes before the starting and screaming took place, so as to break up whatever there was of mere habit about it; and not only to be waked up, but to get out of bed, walk across the room two or three times, and throw back the bed-covering, so as to allow the confined air to escape, then go to bed again, and avoid sleeping any in the daytime. By these means, and these alone, the starting, etc., began to abate, and to diminish in frequency within a fortnight, and in two months with the results already stated.

